# Account of the Diseases Admitted into the Liverpool Dispensary

**Published:** 1801-02

**Authors:** J. Bostock

**Affiliations:** Liverpool


### *To the Editors of the Medical and Phyfical Journal.*

GENTLEMEN,

THE following communication may probably appear to you worthy of a place in your Journal, as exhibiting a concife view of the ftate of difeafe, during the laft year, among the poor of our large and populous town. I am, &c.

*Liverpool, Jan.* 19, 1801. J. BOSTOCK.

*Account*

*Account of the Diseases admitted into the Liverpool Dispensary, between Jan.* 1, 1800, *and Jan.* 1, 1801.

| | | | |
|---|---|---|---|
| Fevers | 2603 | Diarrhœa | 723 |
| Difeafes of the Eyes | 283 | Hyfteria | 99 |
| Sore Throat | 221 | Amenorrhœa | 320 |
| Inflammation of the Cheft | 78 | Convulfions | 32 |
| Confumption | 90 | Infanity | 5 |
| Rheumatifm | 449 | Debility | 356 |
| Eryfipelas | 18 | Dropfy | 107 |
| Small-Pox | 176 | Scrofula | 142 |
| Meafles | 119 | Rickets | 4 |
| Scarlet Fever | 10 | Syphilis | 511 |
| Thrufh | 55 | Jaundice | 48 |
| Piles | 57 | Gravel | 68 |
| Hæmorrhagies | 114 | Worms | 198 |
| Catarrh | 434 | Eruptions and Difeafes of the Skin | 2287 |
| Dyfentery | 383 | Burns and Scalds | 116 |
| Head-ach and Vertigo | 159 | Abfceffes, Ulcers, &c. | 868 |
| Apoplexy | 8 | Wounds, Fractures, and Diflocations | 1013 |
| Palfy | 29 | Cancers | 19 |
| Stomach Complaints | 658 | Ruptures | 137 |
| Epilepfy | 17 | | |
| Dyfpnœa, Cough | 1523 | | |
| Chin-Cough | 39 | | |
| Colic | 102 | Total | 15165 |
| Cholera | 487 | | |

Upon comparing the above Lift with the account of difeafes admitted into the Difpenfary in previous years, the firft circumftance which arrefts the attention, is a very large increafe in the total amount of the patients. The numbers admitted in the years 1797, 1798, and 1799, are 12,162, 12,108, and 13,052, refpectively, which gives an average for the three years of 12,441 per annum. In the year 1800 therefore an increafe has taken place of 2,724, which is above one-fixth of the whole number. To account for fo great an addition, we obferve that the difeafes which are claffed under the head of Fever, confiderably exceed their accuftomed proportion; during the three preceding years the average number of Fevers was 1629; which gives an increafe, in the year 1800, of 974. The caufes of this additional frequency of Fever amongft the poor, may with great probability be referred to the circumftances which have been pointed out by your Correfpondents in the metropolis, as producing the fame effect in the fphere of their obfervations; I refer to a deficiency of food, a want of fufficient cloathing, and great dejection and anxiety of mind. The commercial fpirit of Liverpool has happily prevented its inhabitants from feeling the diftreffes of the times in nearly fo great great a degree as they have been experienced in many other places, yet even here the distress is widely diffused, and in many instances extreme.

The Small-pox and Measles of the year 1800, have been rather below the average of the three preceding seasons.

Under the head of Rheumatism, we find in the year 1800, that the number of cases has been considerably less than usual; the average of the three years being 580, whereas the above list indicates only 449. This diminution is obviously to be attributed to the unusual warmth and dryness of the season; to the same cause we may also ascribe a great diminution of the usual number of Catarrhal complaints, which are this year little more than one-third of their usual proportion.

Another important variation in the list of diseases for the last year, which must also be attributed to the peculiarity of the season, is the unusual frequency of the diseases of the intestinal canal. During this period, 1593 cases appear upon the books of the Dispensary, under the heads of Dysentery, Cholera, and Diarrhœa; which is above four times the average number of these complaints.

The only disease which remains to be pointed out, as affording any thing worthy of remark, is that class of ailments, which are entitled Debility; these, as might be expected, have during the year 1800 almost doubled their usual proportion. They consist in a great measure of old and needy females, who, unable to procure a livelihood by their labour, and chiefly dependent upon precarious charity, drag on a miserable existence, which in the present season of scarcity is rendered doubly wretched.

It may be proper to notice, that Liverpool, besides an Infirmary, contains a Lunatic Asylum, where a great part of the cases of Insanity which occur among the poor, are admitted; and a Charity for Lying-in Women, where Puerperal Diseases are relieved.

*** *The Editors are much obliged to* Dr. Bostock *for the above Communication; they invite him to continue it annually, and will be glad to receive similar Communications from Hospitals and Dispensaries in every part of the kingdom.*

